# Healthy Oral Lifestyle Behaviours Are Associated with Favourable Composition and Function of the Oral Microbiota

**DOI:** 10.3390/microorganisms9081674

**Published:** 2021-08-06

**Authors:** Shirleen Hallang, Anders Esberg, Simon Haworth, Ingegerd Johansson

**Affiliations:** 1 Faculty of Health Sciences, Bristol Dental School, University of Bristol, Bristol BS1 2LY, UK; shirleen.hallang@bristol.ac.uk (S.H.); simon.haworth@bristol.ac.uk (S.H.); 2 Department of Odontology, Umeå University, 901 87 Umeå, Sweden; ingegerd.johansson@umu.se; 3 Medical Research Council Integrative Epidemiology Unit, Department of Population Health Sciences, Bristol Medical School, University of Bristol, Bristol BS8 2BN, UK

**Keywords:** oral behaviour, lifestyle, oral microbiome

## Abstract

Modifiable lifestyle interventions may influence dental disease by shifting the composition of the oral microbiota. This study aimed to test whether lifestyle traits are associated with oral microbiota composition and function. Swedish volunteers, aged 16 to 79 years, completed a lifestyle traits questionnaire including lifestyle characteristics and oral health behaviours. Bacterial 16S rDNA amplicons were sequenced and classified into genera and species, using salivary DNA. Microbiota functions were predicted using Phylogenetic Investigation of Communities by Reconstruction of Unobserved States and the KO Database of Molecular Functions by ortholog annotation. Tests for association used partial least squares and linear regression analysis with correction for multiple testing. The main analysis included 401 participants and 229 common bacterial species (found in ≥10% of the participants). The overall microbiota composition was strongly associated with questions “*do you think caries is a disease?*” and “*do you use floss or a toothpick?*”. Enriched relative abundance of *Actinomyces*, *Campylobacter*, *Dialister*, *Fusobacterium*, *Peptidophaga* and *Scardovia* genera (all *p* < 0.05 after adjustment for multiple testing), and functional profiles showing enrichment of carbohydrate related functions, were found in participants who answered “no” to these questions. Socio-demographic traits and other oral hygiene behaviours were also associated. Healthier oral microbiota composition and predicted functions are found in those with favourable oral health behaviours. Modifiable risk factors could be prioritized for possible interventions.

## 1. Introduction

Healthy humans are colonized by a wide range of commensal organisms which form niche-specific microbiotas. Eubiosis and dysbiosis of these microbiotas are thought to be relevant to an increasing range of health outcomes [1,2]. Lifestyle interventions which modulate bacteria or other microorganisms are, therefore, one possible way to influence disease, but there are few examples where this is used as a major treatment modality in clinical practice.

An exception is in the management of dental diseases, where modulation of the oral microbiota is a main form of prevention for dental caries and periodontitis. Here, the aim is to shift the characteristics of the oral microbiota from dysbiosis to an eubiotic state through a combination of interventions. These interventions include both changes to lifestyle (oral hygiene behaviours, dietary habits, etc.) as well as topical use of antimicrobial agents, such as chlorhexidine in selected cases [1,3]. Thus, dental caries and periodontitis act as model examples of conditions where lifestyle interventions aim to change the microbiota composition or function.

While these interventions are effective in reducing clinical disease burden [3,4], it is not altogether clear which lifestyle factors are most influential in shifting the microbiota characteristics, nor which species and microbiota functions are most strongly associated with these behaviours. For example, flossing has been reported to be associated with altered measures of microbial diversity in some studies [5] but not in others [6], and other studies report only small effects of lifestyle choices [7]. Likewise, several reviews have discussed lifestyle interventions and their effects on single bacterial species or a limited number of species, including modifications to diet, use of tobacco, alcohol consumption and oral hygiene behaviours [1,8], however there are few studies which assess the entire oral microbiota and fewer still have adopted a systematic approach to assess for lifestyle associations with microbiota function [5,7].

It would, therefore, be useful to systematically screen lifestyle factors for association with oral microbiota composition and function, which was the aim of the present study. The results of this may highlight the most relevant lifestyle factors which could be targeted in trials to improve oral health, as well as provide a model to prioritize interventions for other microbiota-related health outcomes.

## 2. Materials and Methods

### 2.1. Study Cohort

The study included 427 participants aged between 16 and 79 years who lived in the northern part of Sweden. Participants <20 years were recruited as they attended their annual dental check-up, and participants aged (≥20 years) were volunteers who responded to a request for study participants. The exclusion criteria were cognitive disability, severe illness, antibiotic treatment within 3 months and inability to communicate in Swedish or English.

### 2.2. Questionnaire Information

The participants completed a questionnaire on living conditions, tobacco use, medical status and medications, lifestyle traits including lifestyle characteristics and oral health behaviours. The core questions were taken from a questionnaire used in the Västerbotten Intervention Programme [9], but were supplemented with additional questions specifically about oral health behaviours and attitudes reflecting oral disease risk factors. The questions and response options are presented in Table S1. Habitual food intake over the latest year was recorded using a semi-quantitative food frequency questionnaire (FFQ) with 93 questions for food items or food aggregates. The response options were “never”, “less than once a month”, “1–3 times per month”, “once a week”, “2–3 times a week”, “4–6 times a week”, “once a day”,”2–3 times a day” and “4 or more times a day”. Participants were asked to estimate portion size using example photographs and select a photograph which matched their regular portion size. For foods which form natural portion sizes (such as an egg) weights were taken from the food database at the Swedish National Food Agency [10]. Reported intakes were transformed to intakes per day. Daily intake of energy, sucrose and sugar (sucrose plus the monosaccharides glucose and fructose) were calculated using weights from the Swedish National Food Agency [10]. FFQ assessed intakes have been validated against 10 repeated 24-hour recalls [11]. Overall diet quality was summarized using a healthy eating index [12].

### 2.3. Microbiota Analysis

Participants were asked not to brush their teeth on the morning of saliva collection and not to eat or drink for 1 h before saliva collection. Stimulated saliva was then collected for 3 min while the participants chewed on a 1g piece of paraffin wax. Saliva samples were stored at −80 °C until used.

DNA was extracted from saliva, a mock community, and a negative control (ultra-pure water). Bacterial 16S rDNA amplicons were generated from the v3—v4 hypervariable region using PCR with fusion primers with 341F (ACGGGAGGCAGCAG) forward and 806R (GGACTACHVGGGTWTCTAAT) reverse primers as described by Caporaso [13]. Equimolar 16S rDNA amplicon libraries were pooled and purified using AMPure XP beads (Beckman Coulter, Stockholm, Sweden) and sequenced using the Illumina Miseq platform. The samples were spiked with 5% PhiX (Illumina, Stockholm, Sweden). Each run included two mock samples and two negative controls alongside the test samples.

Sequence reads were de-multiplexed using deML [14], and cleaned using DADA2 in the QIIME2 next-generation microbiome bioinformatics platform [15,16,17]. During cleaning pair-end reads were fused, primers, ambiguous, chimeric and PhiX sequences were removed, and amplicon sequence variants (ASVs) were retained. These ASVs were then classified against the expanded Human Oral Microbiome Database (eHOMD) [18,19]. ASVs with at least 2 reads and 98.5% identity with a named species or unnamed phylotype in eHOMD were retained, and those with the same Human Microbial Taxon (HMT) ID number were aggregated. The HMT aggregated taxa were standardized to the level of the sample with fewest reads (19,700 reads), and then transformed using inverse hyperbolic sine transformation. This transformation was selected because zero values are common in species-level abundance variables, and this method provides values for variables containing zeros.

The term “species” is used in the remainder of the text to refer to both species and unnamed phylotypes for simplicity.

### 2.4. Prediction of Oral Microbiota Functions from the 16S rRNA Gene Sequences

Predicted molecular functions of the oral microbiota were generated from the obtained 16S rRNA sequences using Phylogenetic Investigation of Communities by Reconstruction of Unobserved States (PICRUSt2) [20] and the KO Database of Molecular Functions by ortholog annotation (KEGG orthologues, KO, within QIIME2) [21]. A closed reference feature table was created using the Greengenes database version 13_5 [22] which is trained against PICRUSt2. Core diversity metrics were estimated in QIIME2, and a KEGG KO feature table was exported for downstream analyses.

### 2.5. Data Handling and Statistical Analysis

Demographic information was summarized as means with standard deviation (sd) for 95% confidence intervals (CI), or by reporting a proportion (%). Nutrient intakes were adjusted for sex, body mass index (BMI) and estimated energy intake using generalized linear modelling. For microbiota comparisons, Shannon and Simpson alpha diversity measures were calculated using QIIME2. All tests were two-sided and *p*-values < 0.05 were considered statistically significant unless false discovery rate correction at FDR 0.05 was applied to account for multiple testing.

PERMANOVA, using Paleontological Statistics (PAST4) [23] with 9999 permutations and FDR-corrected *p*-values, was used to compare groups based on distance measures.

Multivariate analyses used partial least squares (PLS) analysis to identify association between lifestyle traits and measures of microbiota composition and function. Separate models were fitted for abundance measures (relative abundance, restricted to species detected in ≥10% participants) and predicted functions (restricted to functions with a non-zero predicted level in ≥10% participants). Models were fitted using SIMCA *p*+ version 15.0 (Sartorius Stedim Data Analytics AB, Malmö, Sweden) and variables were scaled to unit variance. Cross-validation was performed using a K-fold method, with systematic removal of every 7th observation and prediction of the remaining observations (Q^2^-values). Results of overall model fit and separation were displayed in score loading plots. Importance attributable to different lifestyle traits was plotted as bar plots of the variance explained (R^2^) and variance predicted (Q^2^) by each lifestyle trait in the fitted and cross-validated models. Volcano plots, based on the VIP-value (metric summarizing the importance of each variable in driving the observed group separation) and *p*(corr) (a loading scaled as a correlation coefficient) between the model and original data were used to illustrate the distribution of genus/species or most influential functional pathways. For genus/species, variables were considered influential if they had VIP >1.5 and *p*(corr) <−0.50 or >0.50, and for predicted functions VIP >1.9 and *p*(corr) <−0.65 or >0.65.

Regression models were carried out using relative species-level abundance for species detected in ≥10% participants. Models were fitted for each bacterial species in relation to each lifestyle trait using linear regression and included adjustment for age, sex and educational level. *p*-values were adjusted for multiple testing using the Benjamini-Hochberg method with false discovery rate set to 0.05. As a sensitivity analysis, species detected in <10% of participants were modelled using logistic regression, with adjustment for covariates and multiple testing as described above.

## 3. Results

### 3.1. Study Group and General Sequencing Results

In total, 427 participants met the inclusion criteria, of which 17 were excluded for missing questionnaire information and nine did not have saliva available for DNA extraction, leaving 401 participants in the final study group. Of these, 62.3% were females, 51.6% were below 20 years, and mean (95% CI) BMI was 23.1 (22.8, 23.4), with 24.4% having a BMI ≥ 25 (overweight or obese). In this group, 6.4% reported being current or past smokers and 12.9% were current or former users of Swedish snus (snuff) (Table 1). Further characteristics of the study group are shown in Table 1 and Table 2.

After quality filtering and chimera removal, 20,600,607 sequences remained for the 401 participants and were clustered into amplicon sequence variants (ASVs) which represented 116 genera and 466 species or phylotypes with 2 or more reads when classified against the eHOMD at ≥98.5% identity. The mean (sd) number of reads/sample was 51,373 (17,268) and the negative controls yielded (mean (sd) 141 (52)) reads. The species in the mock communities were correctly identified. For the present paper species/phylotypes detected in ≥10% of the participants were considered for all lifestyle traits in the main analyses (229 common species), and uncommon species (detected in <10%) were only included in species-by-species regression sensitivity modelling. A full list of common species/phylotypes with their relative abundances are shown in Table S2.

### 3.2. Association between Self-Reported Lifestyle Characteristics and Oral Health Behaviours and Overall Oral Microbiota

**Figure 1:**
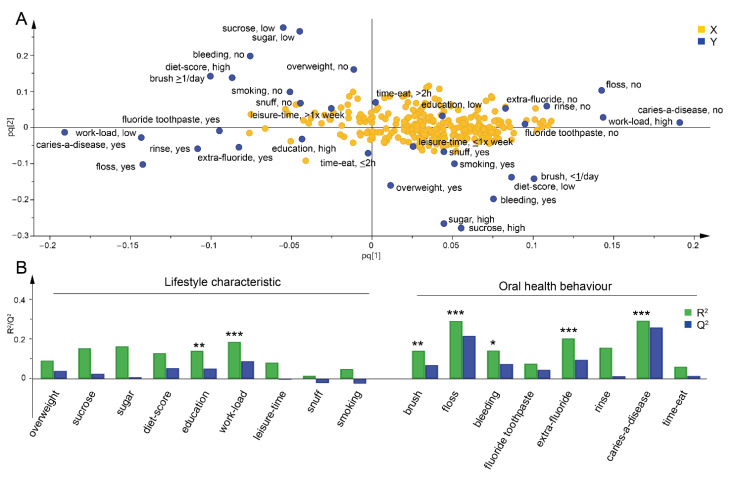
Lifestyle characteristics and oral health behaviours associated with oral microbiota composition. Associations between lifestyle characteristics and oral health behaviours, and saliva microbiota composition were evaluated by orthogonal partial least square regression analysis, where the oral health behaviours and lifestyle characteristics were introduced as dependent variables (y) and bacterial genus and species as independent variables (x). (**A**) A loading score plot of the generated model that illustrates the correlation between the participants’ lifestyle characteristics and oral health behaviours and the composition of their oral microbiota. (**B**) Bar plot show the R^2^ value which indicates the fraction of the original data explained by the model and the Q^2^ parameter indicates the fraction of the original data predicted by the 7-fold cross-validation model. * indicates significant models for indicated trait (P_CV-ANOVA_ * < 0.05, ** < 0.01, *** < 0.001).

To explore the potential effect of self-reported lifestyle traits including oral health behaviours and lifestyle characteristics on overall oral microbiota composition, orthogonal partial least square regression analysis with all selected lifestyle traits (*n* = 17, see Table 1 and Table 2) was carried out. The model illustrates the relationship between and among the bacterial genera/species and the lifestyles traits. The overall model indicated that characteristics associated with higher education, healthy lifestyle, good oral hygiene and use of fluoride products clustered together, seen at the left of Figure 1A. Lifestyle traits that were significantly influential in the model were level of education and physical workload, and with regard to oral health behaviours, tooth-brushing frequency, flossing, use of extra fluoride and whether the participant thought that caries was a disease, were also influential (P_CV-ANOVA_ < 0.05, Figure 1B).

### 3.3. Lifestyle Associations with Single Bacterial Species or Phylotypes

**Figure 2:**
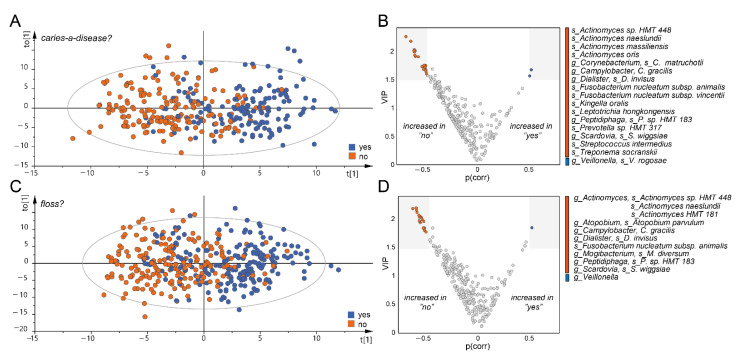
Identification of genera and species which are most strongly associated with two oral health behaviours. Orthogonal partial least square regression discrimination analysis was used to evaluate the association between participants answer to the questions (**A**,**B**) “*do you think caries is a disease?*” and (**C**,**D**) “*do you use floss or a toothpick?”* and the oral microbiota composition. The model score plots (**A**,**C**) illustrate the observed group separation (each participant is represented by a dot) based on their microbiota profile. (**B**,**D**) In order to identify the subset of bacterial species/genus important for the observed group separation, selection based on a combination of Variable Influence in Projection (VIP) and *p* (PLS correlation coefficient) was performed. VIP is a metric that summarizes the importance of each variable in driving the observed group separation and *p*(corr) is a loading scaled as a correlation coefficient (ranging from −1.0 to 1.0) between the model and original data. Inclusion criteria were set to VIP >1.6 and *p*(corr) <−0.5 or >0.5. (g_) indicates genus level, (s_) species level.

The two lifestyle traits most strongly associated with overall microbiota composition were “*do you think caries is a disease?*” and “*do you use floss or a toothpick?*”. To understand which bacterial genera or species were driving this association, we used orthogonal partial least square regression discrimination analysis to evaluate these traits in further detail. The models showed evidence for association at the species level (R^2^ = 47.2%, Q^2^ = 23.0%, (P_CV-ANOVA_ = 1.8 × 10^−17^) and (R^2^ = 35.5%, Q^2^ = 19.7%, P_CV-ANOVA_ = 1.7 × 10^−17^), respectively (Figure 2A,C). The models indicated, to some extent, overlapping enrichment of species belonging to the genera *Actinomyces*, *Campylobacter*, *Dialister*, *Fusobacterium*, *Peptidophaga* and *Scardovia* to associate with answering “*no*”, whereas answering “*yes*” associated with enrichment of *Veillonella* in both models (Figure 2B,D).

**Figure 3:**
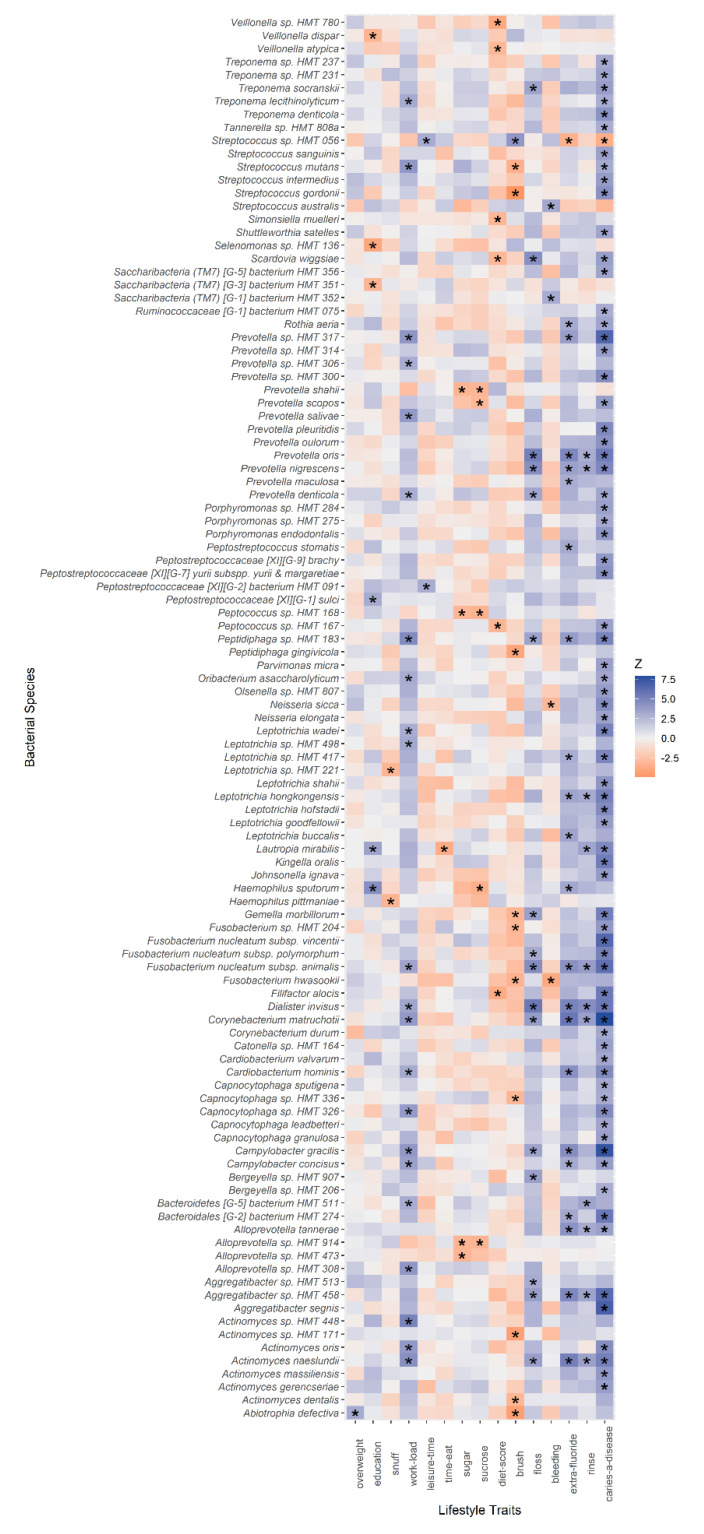
Association between lifestyle traits and relative abundance of common bacterial species. Z scores indicate the strength and direction of association and are obtained from linear regression models. Associations with Benjamini–Hochberg-adjusted *p* values < 0.05 are indicated with (*). All models included adjustment for age, sex and highest educational level.

To further evaluate species associations with oral health behaviours and lifestyle characteristics, we performed sensitivity analysis to adjust for age, sex and highest educational level for all 17 lifestyle traits and the same 229 common species, i.e., present in at least 10% of the participants, included in the multivariate analysis. After adjustment for multiple testing, abundance of 21 species were associated with three or more lifestyle traits and nine lifestyle traits were associated with an abundance of five or more common species (Figure 3). Results were concordant with the main analysis, where “*do you think caries is a disease?*” was the strongest associated oral health trait. Sensitivity analysis of uncommon species did not find any association passing correction for multiple testing. Results are presented in full in Tables S3 and S4.

### 3.4. Lifestyle Characteristics and Oral Health Behaviours Associated with an Overall Change in Predicted Microbiota Functions

**Figure 4:**
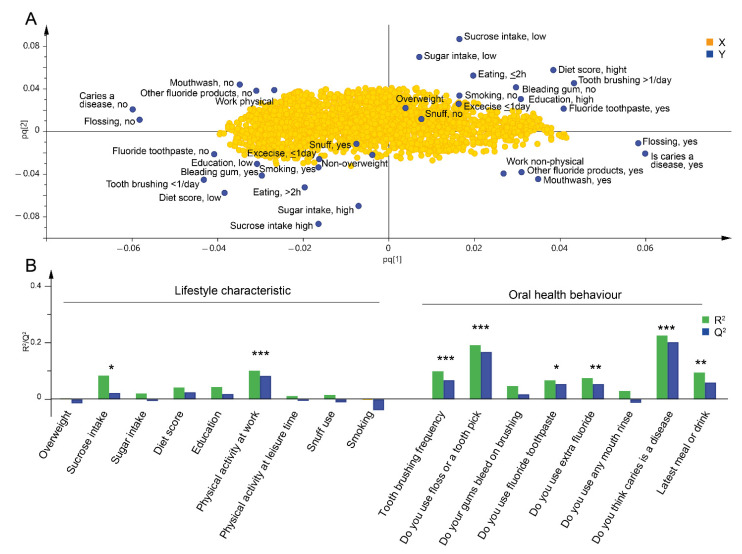
Predicted microbiota functions related to lifestyle characteristics and oral health behaviours. Orthogonal partial least square regression analysis was used to evaluate the effect of participants’ (*n* = 401) oral health behaviours and lifestyle characteristics (*n* = 17 lifestyle traits) on the composition of the predicted microbiota functions (*n* = 10,140). (**A**) Loading score plot of oral health behaviours and lifestyle characteristics as dependent-y variables and predicted microbiota functions as independent-x variables. The loading score plot illustrates the correlation between the y and x variables. (**B**) Bar plot showing R^2^-value (green bar, indicates the fraction of the original data explained by the model) and Q^2^-value (blue bar, indicates the fraction of the original data explained by the seven-fold cross-validation model). * indicates significant difference between the respondents’ answers and the indicated question (P_CV-ANOVA_ * < 0.05, ** < 0.01, *** < 0.001).

Given the associations between lifestyle traits and oral microbiota composition, we hypothesized that there might be associations between lifestyle and healthy or unhealthy microbiota functions. Microbiota functions were predicted using orthologue annotation (KEGG orthologues, KOs) and then modelled in relation to lifestyle traits. The model used functions with a prevalence of at least 10% among the participants (10,140 functions included out of the total number of 10,543 predicted functions). The overall model indicated that lifestyle traits associated with unhealthy oral health behaviours along with unhealthy diet, physical work, lower educational level and not thinking that caries is a disease clustered to the right (Figure 4A). Six lifestyle traits had significant association with predicted functions; *“physical activity at work”*, “*tooth brushing < 1 per day”*, “*do you use floss or a toothpick?”*, *“do you use a fluoridated toothpaste?”*, *“do you use extra fluoride?”* and “*do you think caries is a disease?*” (Figure 4B) (P_CV-ANOVA_ < 0.05).

Having found a global shift in predicted functions based on lifestyle traits, we next tried to identify functions important for the observed group separation for the two most prominent lifestyle traits, *“do you think caries is a disease?”* and *“do you use floss or a toothpick?”.* Orthogonal partial least square regression discriminant analysis was used and based on the top 50 functions for each lifestyle trait and answer option based on a combination of VIP and *p*(corr) (VIP >1.9, *p*(corr) <−0.65 or >0.65), as described previously.

The model using the trait *“do you think caries is a disease?”* explained 29.2% of variance in the original data and was able to predict 20.0% after cross-validation (P_CV-ANOVA_ = 7.2 × 10^−15^). Unique enriched microbiota functions (represented by two predicted genes or more (number indicated in parenthesis after each function)) linked to the answer “*no*” were Amino sugar and nucleotide sugar metabolism (7), O-Antigen nucleotide sugar biosynthesis (4), ABC transporters (4), Carbon metabolism (3), Starch and sucrose metabolism (3), Pentose and glucuronate interconversions (2), Galactose metabolism (2), and Streptomycin biosynthesis (2). Conversely, the answer “yes” was associated with to Propanoate metabolism (2), Aminoacyl-tRNA biosynthesis (2), Arginine and proline metabolism (2), Phenylalanine, tyrosine and tryptophan biosynthesis (2), Glycerolipid metabolism (2), Two-component system (2), and Carbapenem biosynthesis (2).

With respect to the trait *“do you use floss or a toothpick?”*, the model explained 26.6% of the original data and predicted 18.6% after cross-validation (P_CV-ANOVA_ = 2.1 × 10^−16^). Answer “no” associated with unique enrichment in functions linked to e.g., Microbial metabolism in diverse environments (9), Carbon metabolism (5), Fructose and mannose metabolism (5), Carbon fixation pathways in prokaryotes (4), TCA cycle (3), Starch and sucrose metabolism (3), Biosynthesis of cofactors (2), Peroxisome (2), Nicotinate and nicotinamide metabolism (2), Galactose metabolism (2), Glycolysis /Gluconeogenesis (2), Biosynthesis of amino acids (2), Pyruvate metabolism (2) and Nitrogen metabolism (2). Functions linked to “yes” were Biosynthesis of amino acids (10), ABC transporters (5), Phenylalanine, tyrosine and tryptophan biosynthesis (4), C5-Branched dibasic acid metabolism (3), 2-Oxocarboxylic acid metabolism (3), Aminoacyl-tRNA biosynthesis (3), Arginine and proline metabolism (3), beta-Lactam resistance (3), Valine, leucine and isoleucine biosynthesis (3), Glycine, serine and threonine metabolism (2), Carbapenem biosynthesis (2), Cationic antimicrobial peptide (CAMP) resistance (2) and Glycerolipid metabolism (2). This suggests that not considering caries to be a disease as well as not flossing their teeth was linked to a more carbohydrate focused functional profile or their microbiota. For more specifics on enriched functions, see Table S5.

## 4. Discussion

Effective management of dental diseases requires maintenance or re-establishment of a eubiotic oral microbiota [1]. It is therefore important to understand which lifestyle and behavioural interventions are most effective in modulating the composition or function of the oral microbiota. This study used a hypothesis-free method to identify lifestyle factors which are associated with measures of oral microbiota and function. The main finding is that favourable oral health behaviours, including interdental cleaning, were associated with both the overall composition and the predicted functionality of the oral microbiota. Results of this study could be used to understand lifestyle factors to test in clinical trials to modulate the oral microbiota, as well as provide a model for prioritizing interventions for other microbiotas.

The primary analysis was used to screen for oral health behaviours that are associated with the overall microbiota composition and function, but did not provide inference about whether those compositions and functions are relevant to health. Selected oral health behaviours were therefore followed up with detailed analysis of individual species abundance, and predicted functions taking potential confounders into account. This identified that unfavourable oral health behaviours were associated with relatively higher abundance of caries-associated species such as *Campylobacter gracilis* [24] and *Scardovia wiggsiae* [25,26,27] and higher predicted levels of cariogenic functions, including starch and sucrose metabolism [28,29,30], fructose and mannose metabolism, glycolysis, amino sugar and nucleotide sugar metabolism [29,30], galactose metabolism, TCA cycle [30] and pentose and glucuronate interconversions [29,31]. These species and functions are highly relevant for dental caries, where dysbiosis occurs with selective proliferation of acid-producing and acid tolerating species [1,32] and potential demineralization of tooth enamel. In our results, we see a concerted pattern of association with proliferation of cariogenic species and a carbohydrate-focussed predicted functional profile with increased sugar metabolism. Thus, the results suggest that the microbiota in people with unfavourable oral health behaviours is more cariogenic. This needs to be confirmed in future studies relating predicted oral microbiota function to dental disease status, but is in keeping with the current clinical paradigm which aims to create shift in the dynamic oral biofilm by targeted lifestyle interventions [32].

One such intervention which has been advocated is interdental cleaning [33]. Previous studies have shown flossing to result in altered beta diversity [5] and reduced relative abundance of *Streptococcus* species [34]. Non-flossers have also been shown to have an overabundance of bacterial species implicated in caries and periodontitis [35]. In the present study, *“do you use floss or a toothpick?”* was associated with several species thought to be relevant to oral health. People who answered “no” to this question had higher relative abundance of *Actinomyces naeslundii*, as reported previously [35]; however, the present study identified novel associations including higher relative abundance of *Fusobacterium nucleatum subsp. animalis* in people who replied “no” to *“do you use floss or a toothpick?”*. *Fusobacterium nucleatum*, an anaerobe, was originally classed as part of the “orange” complex in subgingival plaque by Socranksy et. al, which is related to disease progression in periodontitis [36]. Flossing has shown to decrease the relative abundance of bacteria found in periodontal disease [35]. A possible mechanism for this has previously been suggested by Burcham et al. [5], who argued that interdental cleaning might disrupt small ecological niches and induce inflammatory responses against oral bacteria on the one hand, while allowing increased relative abundance of less specialized species on the other hand.

One of the most strongly associated questionnaire responses was whether participants felt that caries was a disease or not. This simple question explained a surprisingly large amount of variation in oral microbiota composition and function. This variable is therefore likely to capture not only the degree of importance that participants place on dental caries prevention, but also a range of other attitudes and beliefs which are relevant to dental diseases. Given that this question is simple but predictive of oral microbiota traits, it could potentially be considered as an adjunct to caries risk assessment in clinical settings. Low educational level and physical work (which is a proxy for socio-economic status in Northern Sweden) were also associated with unfavourable microbiota composition, reflecting the situation with clinical endpoints where socio-economic status is a well-known risk factor for dental caries [37,38,39].

In this population, smoking and snus (Swedish moist snuff) use were not strongly associated with oral microbiota composition in the main analysis. This finding differs from previous studies which report that smoking causes proliferation of species including *Streptococcus sobrinus*, *Eubacterium brachy* [40], *Streptococcus mutans* and *Veillonella dispar* [41]. At genus level, the genera of *Atopobium* [42], *Streptococcus* [42,43], *Prevotella*, and *Veillonella* are all reportedly enriched in smokers [43]. The differences in results between these and the present study may be related to statistical power, as tobacco use is becoming rarer over time in Sweden and over 90% of the participants in this study group reported never having smoked. It is also possible that smoking and snus use have somewhat opposing effects on the microbiota which mask patterns of association, as smoking causes a decrease in salivary pH [44] while snus is reported to increase salivary pH during use [45]. Finally, there may be heterogeneity in the effects of different preparations of snus which could further mask associations, depending on the snus pH which can either increase or decrease pH in the mouth [46].

At present, there is little consensus about the required fasting time before donating saliva for oral microbiota analysis. Some authors have used prolonged fasting periods [47,48] and others have used and suggested shorter periods [49,50,51,52]. In this study, time since last eating or drinking was not associated with microbiota composition in the overall model. This may reflect that the measures used were based on relative abundance measures standardized to the number of reads, which are likely to be relatively stable over a short timescale (hours). This suggests that small differences in fasting time are unlikely to introduce substantial bias into microbiota datasets, provided that relative abundance measures are used. By contrast, measures of function based on RNA sequencing are likely to change rapidly over short timescales since transcription occurs more rapidly than cell proliferation. Studies using RNA-based methods may therefore be more sensitive to time since fasting [47] than the DNA based method used in the present study.

The main strengths include the systematic approach to testing for association, the inclusion of predicted functional analysis and the sample size which is relatively large for studies of the oral microbiota. The limitations include the observational design which does not allow for causal inference, and the limited statistical power for some analyses, given that some species or behaviours were uncommon in this group and given the need for strict correction for multiple testing. An additional limitation to consider when assessing the results is the geographic limitations (Sweden) of the study population as the oral health and lifestyle behaviours vary across the world and among various ethnic groups [53,54].

In summary, the study tested for association between lifestyle factors and oral microbiota composition and function, finding that favourable oral health behaviours are associated with healthier composition and predicted function of the oral microbiota. The results of the study could help prioritize the most important health behaviours and modifiable risk factors to target in prevention of dental diseases.

## Figures and Tables

**Table 1:** General and lifestyle characteristics of the participants in the study group.

General and Lifestyle Characteristics	Value	Variable in Analysis and Short Label in Figures
**Women, %**	62.3	
**Age, years, mean (95% CI)**	28.9 (27.4, 30.5)	
**<20 years, %**	51.6	
**BMI, mean (95% CI)**	23.1 (22.8. 23.4)	
**Overweight/obese (BMI ≥25), %**	24.4	overweight
**Highest educational level for age ^a^, %**	47.4	education
**How do you assess your health?, %**		
good	83.3	
not so good	14.5	
**How was your health last month?, %**		
good	89.8	
not so good	10.2	
**Were you ill the week before sampling?, %**		
yes	15.6	
no	84.4	
**Do you take any medicine?, %**		
yes	24.2	
no	75.8	
**Smoking, %**		smoke
present/ex-smoker	6.5	
never smoked	93.5	
**Snuff use, %**		snuff
present/ex- user	13.0	
never used	87.0	
**Physical activity at work, %**		work-load
non-heavy work	75.4	
heavy work	24.6	
**Physical activity at leisure time, %**		leisure-time
<1 time per week	33.4	
≥1 time per week	66.6	
**When was the last time you ate or drunk?, %**		time-eat
≤2 h ago	70.9	
>2 h ago	29.1	
**Sugar intake, g/day, mean (95% CI)**	56.2 (54.3, 58.0)	sugar
**Sucrose intake, g/day, mean (95% CI)**	31.0 (29.9, 32.3)	sucrose
**Heathy diet score, mean (95% CI)**	11.9 (11.5, 12.3)	diet-score

^a^ The question is phrased “*What is the highest education you have completed*”. This is then coded to reflect whether the participant has the highest educational level which is possible for their age. Highest level refers to upper high school, college or university depending on age.

**Table 2:** Oral health behaviour-related characteristics of the participants in the study group.

Oral Health Behaviours	Value, %	Variable in Analysis and Short Label in Figures
Tooth brushing < 1 per day	10.3	brush
Do you use floss or a toothpick, yes?	51.9	floss
Do your gums bleed on brushing, yes?	21.0	bleeding
Do you use a fluoridated toothpaste, yes?	75.1	fluoride toothpaste
Do you use extra fluoride, yes?	15.6	extra-fluoride
Do you use any mouth rinse, yes?	25.6	rinse
Do you think cavities is a disease, yes?	37.8	caries-a-disease

## Data Availability

Microbiota sequences are available at 10.6084/m9.figshare.14748276 and 10.6084/m9.figshare.14748126 and other data are available upon reasonable request and after acquisition of mandatory ethical and other approvals.

## Supplementary Materials

The following are available online at https://www.mdpi.com/article/10.3390/microorganisms9081674/s1, Table S1: List of questions asked in the questionnaire with response options, codings and short labels used in main text figures; Table S2: List of bacterial species prevalence and relative abundance of species present in at least 10% of the participants; Table S3: Linear regression models adjusted for age, sex and highest educational level for lifestyle traits and common species.; Table S4: Lists of traits or species whose relative abundance was associated with one or more species/lifestyle traits; Table S5: Top 50 PLS-DA predicted microbiota functions.
