# Supplementary material for: Healthy Oral Lifestyle Behaviours Are Associated with Favourable Composition and Function of the Oral Microbiota
Source: Microorganisms. 2021 Aug 6;9(8):1674. doi: 10.3390/microorganisms9081674 (PMC8401320; doi:10.3390/microorganisms9081674)
Supplement: Supplementary file 1 [file microorganisms-09-01674-s001.zip › microorganisms-1285633-supplementary/Table S4.pdf]

Table S4a: List of species whose relative abundance was associated with one or more lifestyle traits.

Lifestyle characteristics and oral behaviors (number of Lifestyle traits)	Common species	Code
5	*Fusobacterium nucleatum subsp. animalis*	s141
5	*Actinomyces naeslundii*	s13
5	*Dialister invisus*	s120
5	*Corynebacterium matruchotii*	s112
4	*Campylobacter gracilis*	s80
4	*Streptococcus sp. HMT 056*	s422
4	*Aggregatibacter sp. HMT 458*	s35
4	*Prevotella oris*	s320
4	*Prevotella nigrescens*	s318
4	*Peptidiphaga sp. HMT 183*	s271
4	*Lautropia mirabilis*	s208
3	*Campylobacter concisus*	s78
3	*Alloprevotella tannerae*	s45
3	*Streptococcus mutans*	s414
3	*Scardovia wiggsiae*	s371
3	*Prevotella sp. HMT 317*	s338
3	*Prevotella denticola*	s306
3	*Leptotrichia hongkongensis*	s213
3	*Haemophilus sputorum*	s169
3	*Gemella morbillorum*	s153
3	*Cardiobacterium hominis*	s104
2	*Capnocytophaga sp. HMT 336*	s93
2	*Capnocytophaga sp. HMT 326*	s90
2	*Bacteroidetes [G-5] bacterium HMT 511*	s63
2	*Bacteroidales [G-2] bacterium HMT 274*	s55
2	*Alloprevotella sp. HMT 914*	s44
2	*Treponema socranskii*	s439
2	*Treponema lecithinolyticum*	s434
2	*Streptococcus gordonii*	s410
2	*Rothia aeria*	s351
2	*Prevotella shahii*	s327
2	*Prevotella scopos*	s326
2	*Peptococcus sp. HMT 168*	s273
2	*Peptococcus sp. HMT 167*	s272
2	*Oribacterium asaccharolyticum*	s260
2	*Neisseria sicca*	s250
2	*Leptotrichia wadei*	s229
2	*Leptotrichia sp. HMT 417*	s223
2	*Actinomyces oris*	s15
2	*Fusobacterium sp. HMT 204*	s147
2	*Fusobacterium nucleatum subsp. polymorphum*	s143
2	*Fusobacterium hwasookii*	s138
2	*Filifactor alocis*	s132
2	*Abiotrophia defectiva*	s1
1	*Capnocytophaga leadbetteri*	s86
1	*Capnocytophaga granulosa*	s84

1	*Actinomyces gerencseriae*	s8
1	*Bergeyella sp. HMT 907*	s69
1	*Bergeyella sp. HMT 206*	s66
1	*Actinomyces dentalis*	s6
1	*Veillonella sp. HMT 780*	s468
1	*Veillonella dispar*	s465
1	*Veillonella atypica*	s463
1	*Treponema sp. HMT 237*	s446
1	*Treponema sp. HMT 231*	s443
1	*Treponema denticola*	s433
1	*Tannerella sp. HMT 808*	s430
1	*Streptococcus sanguinis*	s420
1	*Streptococcus intermedius*	s411
1	*Alloprevotella sp. HMT 473*	s41
1	*Streptococcus australis*	s406
1	*Alloprevotella sp. HMT 308*	s40
1	*Simonsiella muelleri*	s390
1	*Shuttleworthia satelles*	s389
1	*Selenomonas sp. HMT 136*	s379
1	*Saccharibacteria (TM7) [G-5] bacterium HMT 356*	s368
1	*Saccharibacteria (TM7) [G-3] bacterium HMT 351*	s366
1	*Saccharibacteria (TM7) [G-1] bacterium HMT 352*	s360
1	*Aggregatibacter sp. HMT 513*	s36
1	*Ruminococcaceae [G-1] bacterium HMT 075*	s354
1	*Aggregatibacter segnis*	s34
1	*Prevotella sp. HMT 314*	s336
1	*Prevotella sp. HMT 306*	s333
1	*Prevotella sp. HMT 300*	s329
1	*Prevotella salivae*	s325
1	*Prevotella pleuritidis*	s323
1	*Prevotella oulorum*	s321
1	*Prevotella maculosa*	s312
1	*Porphyromonas sp. HMT 284*	s297
1	*Porphyromonas sp. HMT 275*	s294
1	*Porphyromonas endodontalis*	s291
1	*Peptostreptococcus stomatis*	s288
1	*Peptostreptococcaceae [XI][G-9] brachy*	s287
1	*Peptostreptococcaceae [XI][G-7] yurii*	s286
1	*Peptostreptococcaceae [XI][G-2] bacterium HMT 091*	s279
1	*Peptostreptococcaceae [XI][G-1] sulci*	s278
1	*Peptidiphaga gingivicola*	s270
1	*Parvimonas micra*	s267
1	*Olsenella sp. HMT 807*	s258
1	*Actinomyces sp. HMT 448*	s25
1	*Neisseria elongata*	s245
1	*Leptotrichia sp. HMT 498*	s225
1	*Leptotrichia sp. HMT 221*	s219
1	*Leptotrichia shahii*	s214
1	*Leptotrichia hofstadii*	s212

1	*Leptotrichia goodfellowii*	s211
1	*Leptotrichia buccalis*	s210
1	*Actinomyces sp. HMT 171*	s18
1	*Kingella oralis*	s175
1	*Johnsonella ignava*	s170
1	*Haemophilus pittmaniae*	s166
1	*Fusobacterium nucleatum subsp. vincentii*	s144
1	*Actinomyces massiliensis*	s12
1	*Corynebacterium durum*	s111
1	*Catonella sp. HMT 164*	s107
1	*Cardiobacterium valvarum*	s105
1	*Capnocytophaga sputigena*	s103

Table S4b: List of lifestyle traits which were associated with the relative abundance of different species.

Number of species	Trait
74	caries-a-disease
22	work-load
22	extra-fluoride
16	floss
11	rinse
11	brush
6	diet-score
6	education
5	sucrose
4	sugar
4	bleeding
2	snuff
2	leisure-time
1	overweight
1	time-eat
